# Self-reported removal and expulsion of the dapivirine vaginal ring: qualitative reports from female ring users and their male partners in the Ring Study (IPM 027)

**DOI:** 10.1186/s12889-024-18795-1

**Published:** 2024-05-31

**Authors:** Cecilia Milford, Hariska Ramlal, Rorisang Mofokeng, Letitia Rambally Greener, Annaléne Nel, Jennifer Smit, Mariëtte Malherbe

**Affiliations:** 1 Wits MRU (MatCH Research Unit), Department of Obstetrics and Gynaecology, Faculty of Health Sciences, University of the Witwatersrand, Commercial City Building, 40 Dr AB Xuma Street, Durban, 4001 South Africa; 2 CAPRISA (Centre for the AIDS Programme of Research in South Africa), University of KwaZulu-Natal, Durban, South Africa; 3 IPM South Africa NPC, an affiliate of The Population Council, Inc., New York, NY USA

**Keywords:** HIV prevention, Vaginal ring, Male partners, Dapivirine, Adherence, Sub-Saharan Africa, Ring removal, Expulsion

## Abstract

**Background/aims:**

The dapivirine vaginal ring is a self-administered, women-initiated, discreet, long-acting HIV-1 prevention option for women. It was found to be safe and effective in healthy HIV-negative women who adhered to product use instructions, and has been approved for use in women aged 18 and older in some African countries. A qualitative study was conducted to explore participants’ and their male partners’ discussions on accidental/purposeful vaginal ring removals during The Ring Study (IPM 027 clinical trial).

**Methods:**

Data were collected via in-depth interviews and focus group discussions with female trial participants and their male partners, from seven research centres in South Africa and Uganda. Data were thematically analysed using NVivo.

**Results:**

More participants reported purposeful ring removals than accidental expulsions. Various factors influenced purposeful ring removal – including individual (discomfort during use/sex and to clean it), partner (to show them, because of discomfort during sex, to test if partners could feel it, and concerns of harm), organisational (doctor’s request), and socio-cultural (rumours about sickness and infertility). Some described their own ring use removal, others discussed why other participants removed their rings.

**Conclusions:**

Vaginal ring adherence is critical to improve and support product efficacy. Counselling on vaginal anatomy, vaginal ring insertion and importance of adherence is important to minimise vaginal ring removal. Couples counselling is also important to facilitate support and long-term vaginal ring adherence behaviour. Understanding factors influencing vaginal ring adherence is important for tailoring and targeting messages to support correct and consistent vaginal ring use as it is made available to the public.

## Background

Trends from the 1980s through to the 2000s have seen an increase in acceptability of the vaginal ring (VR), demonstrating a growing social acceptance for this method as a means of delivering drugs via the female reproductive tract [1, 2]. However, adherence to any VR is dependent on acceptability among end-users which in turn is essential for the effectiveness of the VR [3, 4].

The design of the VR is such that the active pharmaceutical ingredient, embedded in a polymeric matrix, diffuses into the vaginal epithelium [1]. The ability of the VR to bypass gastrointestinal absorption and hepatic first pass metabolism allows for sustained therapeutic levels of drugs in circulation [1, 2, 5]. Compared to the oral route of administration which necessitates more frequent dosing and exhibits more side effects, the VR requires less frequent dosing, exhibits reduced incidences of side effects while producing the same pharmacodynamic effect [1]. The VR also allows women to independently choose their method and consider its reversibility when compared to implants, intrauterine devices (IUDs) and injectables, and contrasted with female condoms, allows for discreteness and is a one-size-fits-most [2, 5].

While there are many advantages to the VR, some users describe discomfort during intercourse, concerns regarding safety and side effects, issues with personal hygiene, and the emotional and cognitive burden on the end-user [1, 5–7]. Concerns about insertion, possible discomfort, pain as result of ring size, dislodgement during sex, and expulsion and slippage have been reported [8, 9]. Myths and misconceptions of the impact of the device on reproductive health and possibilities of negative pregnancy outcomes, in addition to male partner attitudes and disapproval also may affect the uptake and use of the VR [8, 9]. Furthermore, VR non-adherence has been attributed to concerns about use during menses, as well as non-disclosure to male partners [5, 7, 9, 10]. In contrast, partner support and sexual satisfaction or increased sexual pleasure, have been cited as important aspects for continuous VR use [8, 9].

Key steps to overcoming barriers to use and the adoption of a novel technology such as the VR, include participant counselling, education, training, and peer support [1, 3, 5]. Adequate counselling from a trained healthcare worker on the possible side effects and potential risks of any product, demonstrating correct insertion techniques, and providing women with the opportunity to practice on a pelvic model, may build confidence in the use of the product [8]. These are important components that can affect women’s willingness to adopt and use the VR [8]. Additionally, healthcare workers could use counselling as a time to dispel any myths and misconceptions women may have about the product. Several studies report that acceptability and adherence increase with familiarity of use and experience, and compared to other prevention methods, the VR was the most consistently used among women who opted to use it [5, 6]. In addition, because the VR is long acting, it could provide a possible reprieve from forgetting dosage times, thus eliminating the need for daily dosing [8].

Specifically, the dapivirine VR is a long-acting, HIV-1 prevention option for women, it is a women-initiated, self-administered, discreet product. The Ring Study (IPM 027) was a multicenter, randomized, double blind, placebo-controlled safety and efficacy trial of a dapivirine vaginal matrix ring in healthy HIVnegative women, conducted at seven research centres in Sub-Saharan Africa (six in South Africa and one in Uganda). During The Ring Study (IPM 027) the dapivirine VR was inserted 4 weekly, for up to 24 months in healthy, sexually active, HIV negative women (18–45 years of age), and 1959 women participated in the study over 104 weeks. The dapivirine VR was found to be well tolerated and effective in reducing the risk of HIV-1 infection, where the incidence of HIV-1 infection was 31% lower in the dapivirine ring group than placebo ring group [11]. In the ASPIRE dapivirine ring study [12], age was significantly related to a risk reduction in HIV-1 infection, with a lower rate of HIV-1 acquisition in women over 21 years, who used the dapivirine ring. The lower level of HIV infection risk reduction among younger women may have been due to physiologic differences in the genital tract, more frequent vaginal or anal sex, lower adherence to VR use or a combination of these factors [12].

After ASPIRE and the Ring Study (IPM 027), the HOPE study (HIV Open-label Prevention Extension, MTN-025) and the DREAM Study (IPM 032) were conducted. These two studies were open-label studies which enrolled former ASPIRE and Ring Study (IPM 027) participants, and provided additional safety and use data on the dapivirine VR [13]. HOPE and DREAM results together suggested that HIV incidence was reduced by about half when the dapvirine VR was used [13].

Following these clinical trials, the dapivirine VR received a positive benefit-risk opinion for the prevention of HIV-1 from the European Medicines Agency (EMA) in July 2020 [14]. This provided the foundation for approval of the VR by the South African Health Products Regulatory Authority (SAHPRA) in 2022, for use in women aged 18 and older, and was in cooperation with the WHO prequalification process conducted in November 2020 [15]. The dapivirine VR has also been approved for use elsewhere, including other African countries, Zimbabwe, Zambia, Uganda and Kenya [16]. Real world use and studies have shown the dapivirine VR is as effective as oral PrEP, and is a preferred PrEP product by some women [17].

Adherence to PrEP products remains a challenge for the efficacy of these products. Non-adherence to microbicides in clinical trials has been identified as one of the major reasons behind the failure to prove efficacy and effectiveness of such products as an HIV prevention method [18–20], and causes efficacy results to be reduced, especially where there is under-reporting of non-adherence. One study comparing daily oral PrEP use with the dapivirine VR demonstrated similar adherence in both groups (57%) [21].

In order to determine efficacy of the VR, it was essential for participants in the dapivirine VR studies to use it as instructed and to demonstrate adherent ring-use behaviour. In The Ring Study (IPM 027), adherence was measured by calculating concentrations of residual dapivirine levels in returned VRs [11]. In addition, self-reported accidental expulsions and purposeful removals were recorded. Accidental expulsions were most commonly reportedly associated with defecation or urination, and common reasons for purposeful removals were to clean the VR, discomfort/pain or on request of the partner, with a few reported removals because of menses [11]. There was often reinsertion after removal in this study, with the duration of removal being limited to a couple of hours rather than days [11]. Although there is existing data on factors influencing adherence, there is a need to further understand and explore multilevel contextual factors that influence and inhibit consistent VR use [22]. This is critical to inform counselling messaging with the introduction of the dapivirine VR.

A qualitative socio-behavioural study was conducted at the Ring Study (IPM 027) research centers to explore participants’ and their partner’s experiences with and opinions about the VR. In this manuscript we specifically describe The Ring Study (IPM 027) participants’ and their male partners’ discussions on accidental VR expulsions and purposeful VR removals. Exploring these discussions provides insight into various factors influencing VR use and adherence, as well as understanding of messaging around VR use, which could impact on adherence and acceptability of the dapivirine VR in the general population.

## Methodology

In-depth interviews (IDIs) were conducted with female Ring Study (IPM 027) participants and their male partners, and focus group discussions (FGDs) were held with female participants, across the seven research centers in South Africa and in Uganda. They were purposively selected for IDIs and FGDs, and female participants who had reported VR removal or expulsion, as well as some who had not reported either, were invited to participate.

There were two rounds of qualitative data collection during The Ring Study (IPM 027), which was conducted from 2012 to 2015. Round 1 of data collection consisted of individual IDIs conducted at each research center. Round 1 IDIs were conducted approximately 6 months to a year after enrolment commenced at each of the different research centres. Approximately 6–10 individual IDIs were planned with trial participants, and 6–10 IDIs were planned with male partners at each research center.

Round 2 of data collection was also conducted at all active research centers, and consisted of FGDs, which were conducted with groups of female participants, and IDIs, which were conducted with male partners. Round 2 of data collection was conducted after last product use visits (after the VR was removed and product discontinued). Round 2 data collection was conducted approximately 14–19 months after Round 1 IDIs at the research centers. Approximately 2–3 focus groups were planned with participants and 6–10 individual IDIs with male partners, at each research center.

Male partner participants were not restricted to partners of women who participated in the IDIs or FGDs, but were partners of any female participant enrolled in The Ring Study (IPM 027). No couples IDIs were conducted. IDIs and FGDs were conducted by clinic/site staff, including research nurses, study counsellors and community teams, who had been trained in qualitative data collection. Most interviewers were matched by gender with the participants, although a few male IDIs were conducted by women interviewers. The interviews and discussions were conducted in participant language of choice (including Afrikaans, Sotho, Tswana, Zulu, Sepedi, Xhosa and Lunganda), depending on site location.

### Ethical considerations

The study was approved by relevant local ethics committees, including Pharma-Ethics (Ref: 11,074,447), University of the Witwatersrand’s Human Research Ethics Committee (Wits HREC; Ref: 110,810), Medical Research Council/Uganda Virus Institute Research Ethics Committee (MRC/UVRI REC, Ref: GC/127/13/03/33), University of Cape Town Human Research Ethics Committee (UCT HREC, Ref: 273/2013) and University of Pretoria Research Ethics Committee (UPREC, Ref: 388/2013). All female participants provided separate written informed consent to participate in the qualitative research component. They were advised that non-participation in this study component would not affect their participation in the clinical study. Female participants were invited to refer their male partners for an interview. Male partners provided separate written consent for participation.

### Data analysis

**Fig. 1:**
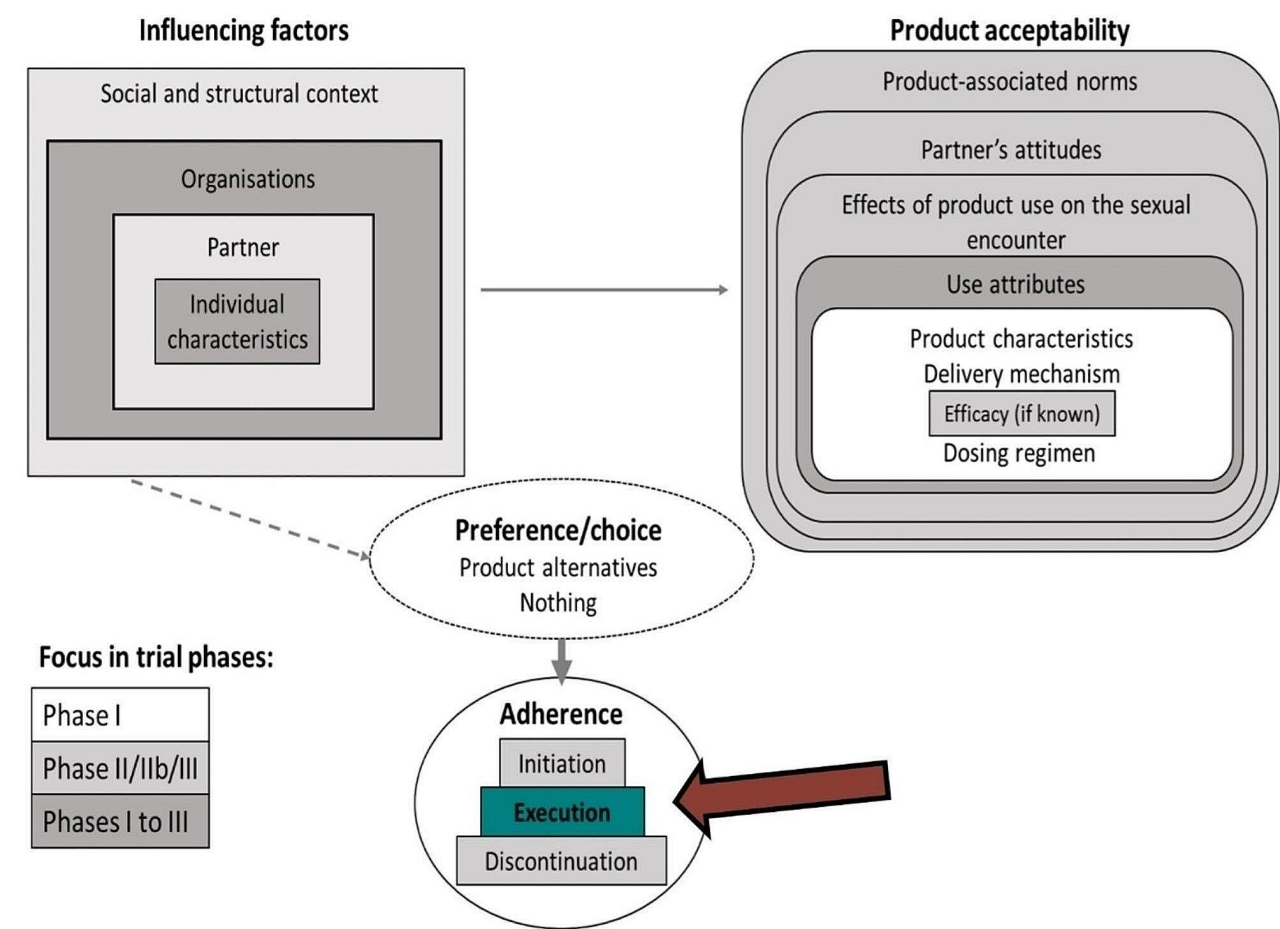
Framework of adherence and factors influencing adherence [18]

All IDIs and FGDs were audio recorded, transcribed, and translated into English. An independent transcription company transcribed and translated all audio files, which were then reviewed for accuracy by representatives from the relevant research centers. The study sponsor conducted a final review to ensure all identifiers were removed from the transcripts for participant confidentiality. A subset of finalized transcripts were read independently by two socio-behavioural researchers (CM, LRG) who were not working on the clinical trial, in order to identify preliminary themes and codes for analysis. Based on the reading of the transcripts, the two socio-behavioural researchers met, discussed, and merged their preliminary codes to create a single draft codebook, incorporating a combination of *a priori* and emergent codes. Codes were generated iteratively with input from questions in the interview guides, emergent themes, and guided by a theoretical framework [23] (see Fig. 1).

According to this framework, adherence is operationalized as: initiation (product uptake or not), execution (whether the product is taken/used as directed or not) and discontinuation (stopping product use). In this study, adherence is largely discussed in terms of execution (whether it is taken/used as directed or not), and therefore results focus on this aspect of adherence. According to the framework, various factors, including individual, partner, organizational and social levels, influence adherence. Participant responses and discussions have been organized under these broad thematic areas. Product acceptability as described in the theoretical framework is not explored in this manuscript.

A qualitative data analysis software program, NVivo (version 10, QSR International) was used to organize, code, and facilitate analysis of the data. Using the draft codebook, a portion of transcripts (about 25%) were double coded by the same two socio-behavioural researchers (CM, LRG), who then discussed any discrepancies and reached an agreement on code definitions, before finalising the codebook, coding the remaining transcripts and analysing the data.

This manuscript focuses on data related to the thematic area “execution: product adherence”. Descriptions of product removal (purposeful) and expulsion (accidental) are described and explored as part of product adherence, and are described according to the various influencing factors (individual, partner, organizational and social).

## Results

The number of IDIs and FGDs conducted with male and female participants per round of data collection, and per research center, are highlighted in Table 1. There were 55 female participant and 46 male partner IDIs conducted in Round 1 across the seven research centers. In Round 2, 18 female participant FGDs (with a total of 147 female participants) and 45 male partner IDIs were conducted. Overall, more male IDIs were conducted than female IDIs, since in Round 2, female data were collected via focus group discussions. Individual demographic data were not collected as part of this qualitative study, but demographic details of all participants in the clinical trial are reported elsewhere [11].

**Table 1:** Number of IDIs and FGDs conducted across the research centers (*n* = 7) per round

Site	Round 1		Round 2			
	IDI type	No. IDIs per site	IDI Type	No. IDIs per site	FGD type	No. FGDs per site (n = no. FGD participants)
**A**	Female IDI	10			Female FGD	3 (*n* = 35)
	Male IDI	3	Male IDI	10		
**B**	Female IDI	7			Female FGD	3 (*n* = 21)
	Male IDI	6	Male IDI	8		
**C**	Female IDI	10			Female FGD	3 (*n* = 27)
	Male IDI	10	Male IDI	10		
D*	Female IDI	10			N/A	N/A
	Male IDI	10	N/A	N/A		
**E**	Female IDI	6			Female FGD	3 (*n* = 20)
	Male IDI	6	Male IDI	6		
**F**	Female IDI	6			Female FGD	3 (*n* = 20)
	Male IDI	5	Male IDI	5		
**G**	Female IDI	6			Female FGD	3 (*n* = 24)
	Male IDI	6	Male IDI	6		
	**Total Female IDIs (Round 1)**	**55**			**Total Female FGDs (Round 2)**	**18 (** ***n*** ** = 147)**
	**Total Male IDIs (Round 1)**	**46**	**Total Male IDIs (Round 2)**	**45**		

*Site D did not participate in Round 2 data collection

Participants’ reports of accidental ring expulsion and purposeful ring removal, and factors influencing these (individual, partner, organizational and social – as applicable) are described in detail below.

### Accidental vaginal ring expulsion

Although most male and female participants felt that it was impossible for accidental VR expulsion to occur, some expressed that initially they did have concerns that the VR might come out accidentally. Across the seven research centers, only a few participants in the qualitative research (*n* = 26) reported that they had experienced an accidental ring expulsion during The Ring Study (IPM 027). There were no major differences in reports of accidental ring expulsion between sites. The reported expulsions occurred in a number of different scenarios, and in some cases more than once. The majority reported that accidental expulsion occurred during, or as a result of, sexual intercourse.

*Participant (P): Yes, it came out by mistake; we were having sex with my partner. Then I came here the next day to report that the ring had come out by mistake.*

*Interviewer (I): I would like [you] to explain in detail how it came out by mistake. Where was the mistake?*

*P: The mistake was that we were having sex and it came out. […] It just came out. I didn’t see. I only saw it on the bed. (Site G, Round 2, Female FGD)*

*P: [A]fter being with my client in a lodge, I do not know what happened but the condom slipped off and remained in me. So when he tried to pull it out it came with the ring! (Site E, Round 2, Female FGD)*

Many also described that accidental ring expulsion occurred whilst they were on the toilet, or during bathing.

*P: [M]ine did come out by accident once. […] That time, so I had a constipated stomach. […] And the time when I thought I could push it back again, it already fell out. […] In the toilet pot. (Site C, Round 1, Female IDI)*

*P: The ring once came off I didn’t know what happened because I had not felt that the ring was out. I came here to the clinic and the doctor did not find it. When I went back home, it means it came out when I was taking a bath. I threw it out with bath water but I found it. There were no problems. (Site G, Round 1, Female IDI)*

However, one participant was not sure how her ring had been expelled.

*P: It did come out and I phoned and reported and they said they would come to fetch me the next day, and then when I came they inserted another one.*

*I: When it had come out, what did you do with it?*

*P: I didn’t see it where it came out.*

*I: It got lost?*

P: yes. (Site A, Round 2, Female FGD)

### Purposeful vaginal ring removal: Self-reported behaviour

More participants reported purposeful VR removals than accidental expulsions (*n* = 30 versus *n* = 26). There were a few participants from all research centers (except sites D and E) who reported a purposeful VR removal. The majority of the purposive VR removals were reportedly for a short period only, with participants replacing their vaginal ring, or getting research center staff to replace the VR, shortly after removal. Some factors led to repeated removals of the VR (e.g. to clean, discomfort during sex), whereas others were one off removals (e.g. to show partner what it looked like, for hospital visit). A variety of influencing factors reportedly resulted in VR removal – individual, partner and organizational level factors.

#### Individual level factors

Purposeful VR removal was influenced by individual participant factors in some instances. The majority of Ring Study (IPM 027) participants reported purposive VR removal during bathing or to clean their ring. Some also described that they took it out to clean during their menses.

*P: I had taken it out to wash it. (Site A, Round 1, Female IDI)*

*P: There was a difference for me because I felt pains in my vagina when I was menstruating. So when I bathed I took out the ring to be able to wash out the dirt. And then I inserted it again.*

*I: Did you take out the ring every time when you had your menses?*

*P: Yes, when I bathed so that I can wash my vagina with my finger to wash out the dirt, and then inserted the ring back again. And I had my menses for about a month or 3 weeks. (Site F, Round 2, Female FGD)*

A few participants described that they purposively removed their VRs due to discomfort experienced during sex or everyday VR use. Some removed it to check it was still in place, since they couldn’t feel it when inserted.

*P: It once…like it came down and I didn’t know, I didn’t understand what they [research center staff] said, it came down and I just removed it. I told myself that maybe it came down. […] And I just removed it, […] I didn’t understand what they said, and I came here. […] And then I explained to them and they explained that if it comes down I shouldn’t remove it but rather push it back. […] …it was after we’ve had that thing [sex]. (Site C, Round 1, Female IDI)*

*P: I found it difficult to have it inside me [in the beginning]. When I started [the study] I removed it and placed it aside. But it was only for a month. The following months and weeks I was putting it [inside]. I did not feel anything. I even forgot that it was inside. […] When I started, I took it out because I was scared of it. Maybe for two days because I was scared of it. (Site C, Round 1, Female IDI)*

One participant in The Ring Study (IPM 027) removed her VR for fear that it might fall out when she was sick with diarrhea.

*P: I removed it because I can’t remember what happened. I was in the toilet, I don’t know if I had a runny tummy or something, I felt like it was coming out and I removed it to stop it from falling into the toilet. [….] but they were angry with me that I shouldn’t have taken it out. I told them that I was afraid that it would come out because I had taken something to clean my tummy. (Site C, Round 2, Female FGD)*

#### Partner level factors

Partners were identified as influencers in ring removal behaviours. Some participants of The Ring Study (IPM 027) described that they had removed their VRs to show their male partners, often as a once off curiosity.

*P: Because by the time I met my partner I was already using the ring. […] It was ring study, he never noticed, it’s me who told him long after we met that I have something here. […] Yes, I took it out to show him and then I inserted it again. (Site B, Round 2, Female FGD)*

Similarly, some male partners reported requesting that their female partners remove their VRs so that they could see what they looked like.

*P: No I asked myself that this thing [referring to the VR], do you mean this thing can actually do the job that they … the purpose of this research, how? […] It was about two weeks or so, two weeks after she had inserted it, or a month or so. Then I said “no man, let me see this ring”, you see, she said “no, you take it out”. Then I took it out and looked at it. (Site C, Round 1, Male partner IDI)*

One participant of The Ring Study (IPM 027) noted that her male partner wanted to see her ring, so she only showed it to him after consultation with research center staff:

*P: He complained saying he wanted to see it. […] I told him that I do not have right to take it out from the house, I have to take it from where it was put on [at the research center]. If you want to see it let us go together. Even now I was helped by you about what you were explaining to him. [Name of staff member] used to say “you should take it out for him to see before there’s an argument, take it out for him and say this is the thing”. […] Me, I just take it out and show him so that he won’t think this is the snake, and end up saying I am bewitching him. (Site B, Round 2, Female FGD)*

Some female participants reportedly removed their VR because their male partners had requested they remove it, because of discomfort during sex, and this was reiterated by male partners.

*P: I had a problem with that because my partner had a problem so every time when we had sex he wanted me to take it out, until we received counselling. (Site C, Round 2, Female FGD)*

*I: Did you ever ask her to take it out?*

*P: Yes, when it was hurting me, like sometimes I would tell her “this thing is hurting me, we cannot continue”. [….] Sometimes she agreed, and sometimes she refused. (Site C, Round 1, Male partner IDI)*

One female participant described that she had removed her VR to test if her male partner could really feel it during sex. A couple of male partners also reported that they had asked their female partners to remove the VR to see if there was any difference during sex.

*I: Why were you asking her to remove it?*

*P: I wanted to tell if there would be a difference. (Site E, Round 2, Male partner IDI)*

One male partner described that he had requested his female partner to remove the VR because of his concerns that it could cause harm. Similarly, a female participant described how her partner had concerns that the VR would harm him. He forcibly removed her VR after she had told him that she had quit the study, and he had found out that this was not true.

*P: I thought that it was dangerous, that it could cause harm. (Site E, Round 2, Male partner IDI)*

*P: And he told me that, “that ring of yours makes me sick” that it makes his penis sick. So I brought him here to the clinic and he was treated. We had a fight and he refused me to go back and told me to return their papers [refers to Participant Information Sheet]. I got the papers and kept them somewhere else and I informed him that I had quit. He remained convinced that I had quit and I never said a thing about it. He later heard that I was still active in the study and came back telling me that it still hurts his penis. (Laughs) […]*.

*I: What happened after he complained again?*

*P: I told him that I quit but he forcefully got a hold of me and removed the ring from my vagina and he kept it then I left him. (Site E, Round 2, Female FGD)*

However, the majority of male partners interviewed explicitly stated that they did not ask their female partners to remove the VR. In addition, one described how his partner had offered to show him the ring but he did not want to see it.

*P: She offered to take it out, I then said what if I don’t feel right after seeing it? […] What will happen? If I lose interest in her? So then she decided to let it stay inside her. (Site B, Round 2, Male partner IDI).*

Some female FGD participants reported that they did not remove their VRs upon their partners’ requests, and this was reiterated by some male participants.

*P: My man touched it and asked me what I had put in the vagina, (others laugh) he told me to remove it, but I refused and I told him that if he wants me to remove it, let us give it up. He ended up ignoring it, but I never removed it. (Site E, Round 2, Female FGD)*

*P: I once asked her to remove it, and she refused. She said if she removes it, she would be infected with HIV if sleep around with multiple partners. If the ring is inside, her safety is guaranteed. (Site F, Round 2, Male partner IDI).*

#### Organizational level factors

Organizational level factors include external factors such as workplace, communities and healthcare facilities. A few participants of The Ring Study (IPM 027) reportedly removed their VRs when they were unwell or because of a doctor’s request.

*P: I once took it out when I was going to the hospital for treatment, and I inserted it back in when I came out. (Site C, Round 2, Female FGD)*

*P1: I did take it out but it was the doctor who had said so, I had severe cervical pains, and I phoned him and he said I must take it out and put it in the packets which we are given here. They came to fetch me and the doctor checked what was wrong and then he put it back. […]*

*P2: Yes, I also did take it out, it was the doctor who said I must take it out because when they checked me the result, the pap smear test did not show anything and they referred me to the hospital in [name of town] to take a sample. (Site G, Round 2, Female FGD)*

One participant reported that she had removed her VR as she was concerned about the ring expiry date.

*P: The reason why I took it out was because I missed my [visit] date and I was not going to be able to come here [research center]. So,… I don’t know if I can say that it properly explained to me the problem when you have missed your date. […] So, I thought to myself that it was going to expire or something […] (Site G, Round 1, Female IDI)*.

#### Purposeful ring removal: Behavior of others

The Ring Study (IPM 027) participants described various reasons that other participants removed their VRs. Some reported that others had removed their VRs and only reinserted them on their return to the research center, citing strategies for hiding this non-adherent behavior, including staining their rings to make them look used.

*P: Someone else said that she puts it [her vaginal ring] in the tea, and then put it on when she comes here. I said, “really?” She said she puts it in the tea and then it changes [colour], it changes [colour]. (Site A, Round 2, Female FGD)*

#### Individual level factors

A variety of individual level reasons were reported for others’ VR removal – including the possibility of lending rings to other participants of The Ring Study (IPM 027).

*P: There was also someone who I knew who was wearing this ring and I don’t know when she removed it, and it happened that it got lost. Then she came to me asking me to borrow her mine [All laughing], and I said to her “how can I lend you my ring?” She said she was coming to the study the following day. So, I felt that it was not possible for me to lend her my ring because she was going to insert it in herself and when she came back I would also insert it in my vagina, we would both get sick. So, I felt that it was not possible for me to lend her my ring. (Site C, Round 2, Female FGD)*

Other reasons cited for VR removal were that some participants were not committed to the study outcomes, did not think study staff would notice, or that they were enrolled only for the money.

*I: Why do you think these people remove it?*

*P: No, it is people not being committed to what they are up to. Their hearts are not committed that they are not interested. It is the reason why they come and I think they come like for fun and then when they reach there [home] and remove it. (Site E, Round 1, Female IDI)*

*P: I think they removed the ring because they thought it would not be noticed when they came to the study. (Site C, Round 2, Female FGD)*

*P: Others do it [remove the VR] because they are just being naughty. […] They do it because they get money you see. (Site G, Round 1, Female IDI)*

Others reported that other participants removed the VR because it was uncomfortable.

*P: I think it was uncomfortable for them and they were not able to endure it. (Site C, Round 2, Female FGD)*

#### Partner level factors

Some participants described fear of partner reaction, especially when ring use had not been disclosed, as a reason why other participants may have removed their VRs.

*P: My neighbour told me that, “eish, I’m scared that my child’s father might feel the thing they inserted in me there. When I go home I take it out.” (Site C, Round 2, Female FGD)*.

*P: There’s one who has not told her partner…that she is wearing the ring. She takes it out when she goes to [visit] him. And then puts it in when she comes back. She might wear it when she comes back from him. […] That one has not told her partner. (Site G, Round 1, Female IDI)*

There were also reports that others who had disclosed use, removed their VR because their male partners did not like it.

*P: The one who told me said her partner didn’t like the ring; so when she went to him she took it out. (Site G, Round 2, Female FGD)*

#### Socio-cultural level factors

Rumours of participants dying or falling sick, as well as myths that the VR could cause infertility, were also described as reasons for other participants removing their VRs.

*P: I heard some ladies saying that they took it out, trying to scare us. They said they heard that someone who had been coming to the study had died, or something like that. And they said that they didn’t want to get sick, that the research people give us sicknesses that we would never have gotten. So, they said that they took the ring out and put it somewhere and only inserted it the morning of their visit. (Site C, Round 2, Female FGD)*

## Discussion

In this exploratory study, the number of self-reported VR expulsions and removals were low, even though some participants who had reported expulsion/removal were purposively selected to participate in the IDIs/FGDs. Although participants described both accidental expulsion and purposeful removal of the VR, more participants reported purposeful removals, highlighting the importance of adherence counselling. Expulsions could be minimised with ongoing counselling/training on correct insertion technique and are likely to decrease over time with experience and familiarity with use [5, 8]. Some of the reported purposeful removals were repeated (albeit temporary), and others were once off VR removals, as has been found elsewhere [22].

Various factors, such as individual user experience, led to decreased ring removal over time, for example, discomfort with VR use often decreased over time, while the overall satisfaction in the product increases over time [8], resulting in fewer VR removals with time. The intermittent use of the VR – removing it repeatedly but temporarily – could also possibly be related to misunderstanding of the concepts of ‘adherence’ versus ‘consistent use’ (a “complimentary interpretation”), whereby participants may perceive themselves to be adherent [9]. Although participants were counselled on consistent VR use and adherence, it is possible that they could have misunderstood this. Improved awareness of these misunderstandings can assist in developing better defined counselling strategies on the concepts of consistent use and VR adherence, making reference to intermittent use as inconsistent VR use behaviour. Counselling should provide details on level of protection provided with intermittent VR use [7] as well as to highlight partial efficacy and how study product works [24] in order to improved VR use and adherence. In addition, an understanding of real world use, for example concerns around cleanliness and curiosity around removals could be incorporated into the counselling provided.

Participants in this study described various factors that influenced purposeful VR removal, as has been described elsewhere [23]. Firstly, individual factors resulting in ring removal included discomfort of the ring, removal to clean the VR or during bathing, and removal because of menses. In many instances, removal to clean the VR is due to women having a poor understanding of vaginal anatomy and how the vagina works, and that the VR does not get “dirty” with use. Similar individual level factors have also been found to influence uptake and use in previous studies [3, 7, 9]. In order to address discomfort with use, understanding of vaginal anatomy [25], together with demonstrations of insertion and removal of the VR using pelvic models could result in appropriate insertion, increased comfort and hence improved adherence. Furthermore, counselling should include instructions on what can be done if VR users experience discomfort, and on how to reinsert the VR if it is expelled [25]. Although participants in this study did not mention removal because of VR side effects, it has been described in other studies [7] and counselling on possible side effects which could influence adherence is important [25].

Removal of the VR to clean it and allow for menses “flow” has also been reported elsewhere [7, 9]. Other research has highlighted that the socio-cultural context of menses in some countries (e.g. Zimbabwe and South Africa) could evoke feelings of disgust, dirt and shame, which could impact on VR use [10]. This has important implications for counselling on VR use and adherence. Recommendations for concerns regarding hygiene can be addressed through appropriate health information and counselling [7], or may be addressed by allowing women to briefly remove the VR for cleaning, similar to cases of expulsion where it is recommended that the VR is washed prior to insertion [6].

Participants also suggested that some individuals were not committed to study outcomes and were possibly enrolled for the study reimbursement, and therefore did not adhere to VR use instructions. This has been described in other research [9], indicating a need to further explore the impact of reimbursement on study participation and study product use practices. However, it is likely that if someone opts to use a VR in non-study setting, their behaviour will not be influenced by reimbursement.

The partner level factors which led to VR removal included partner request/demand for removal, fear of harm from the VR (or safety concerns), once-off curiosity to see the VR, and/or female participants wanting to test if their partners could actually feel the VR during sex. Similarly, previous research has highlighted that partner attitudes and disapproval have influenced uptake and use of VRs [8], and that non-disclosure of VR use has also led to VR non-adherence [5]. It has been suggested that the maintenance of sexual relationships is more important than VR use guidelines, and that removing the VR for a male partner, especially during sex, minimizes any fears or actual disturbance to a relationship or pleasure during sex [9, 26]. It is therefore critical to understand individual relationship dynamics and to tailor counselling on consistent VR use within partnerships, in order to facilitate VR adherence. Counselling could include developing strategies for retaining the VR during sex, and if the male partner feels the VR, and could include role play of possible discussions with sex partners [25]. Where possible, couples counselling should be considered to increase partner support of VR use.

Participants also described organizational and community level factors that led to VR removal, these included VR removal when unwell, or because of doctor’s requests. Participants described how myths and rumours, for example about safety and future pregnancies, had resulted in participants removing their VRs. It is important to further explore and understand community level and socio-cultural factors, such as attitudes to intravaginal products, myths and rumours and how these may impact on VR removals and long-term adherence behaviour [25], for both VRs and other HIV prevention products.

In The Ring Study (IPM 027), staff provided additional adherence counselling to all participants who reported accidental or purposeful VR removal, in many cases preventing further non-adherent behaviours. In the introduction of the dapivirine ring, which is now being explored in implementation studies, it will be important to ensure that adherence is appropriately defined and understood by potential ring users. VR adherence will result in improved efficacy of the product, enabling greater protection against HIV infection. The VR also provides an opportunity for provision of a multi-purpose prevention technology. Appropriate and targeted counselling, education and peer support [3], as well as providing product choice, with a menu of possible available HIV prevention products, will be critical to reduce barriers to use and facilitate ongoing adherence for increased HIV protection.

### Limitations of the study

This qualitative study was conducted with a subset of clinical trial participants – qualitative studies are exploratory, and data is not generalizable, therefore it is not representative of the entire clinical trial sample. However, it enables in-depth understanding of ring use behaviour.

It is important to note that some interviewers were also clinical trial staff, including research nurses and counsellors, and that this could have contributed to social desirability bias in the interviews. However, in spite of this, participants were able to describe their own and others’ VR expulsions and removals.

Male partners were not restricted to partners of women who participated in the IDIs or FGDs, and no couples’ data were analysed, resulting in a possible lack of information on the importance of couples counselling. In addition, male partners that were interviewed were aware of their female partner’s study participation and VR use, so their views may not reflect those of males who were not aware of their female partner’s VR use. As a result, the information provided by male partners may be biased to reflect more informed views of males who accepted their female partner’s VR use.

In this qualitative study, self-reported expulsions/accidental removals were not confirmed with residual dapivirine results, therefore discussions on ring removal/expulsion are not verified against actual adherence data. However, it is important to understand participants perceptions and descriptions of VR removal to facilitate future education/counselling on VR use.

Participant ages and other key demographic details were not recorded, therefore there cannot be any discussions of possible trends in removal or expulsion related to factors such as age.

Although many participants in this study discussed their own VR removal behaviours, some reported on the actions of “other” participants. This kind of reporting, respondent independent data, may occur when individuals report on behaviours of others to indirectly report their own behaviours, especially when there is sensitivity to reporting of information [27]. Results reporting on other participants’ behaviours should be considered in the light of this.

Finally, the study was conducted some time ago. However, the dapivirine vaginal ring has recently been approved for use in South Africa and other countries. Findings from this study are still relevant and are important for consideration, especially when the VR is made available to the general public.

## Conclusions

VR adherence is critical to improve and support product efficacy. This study provides detailed discussions of reasons why participants in The Ring Study (IPM 027) removed their VRs (dapivirine or placebo rings) and did not fully adhere. Understanding the continuum of adherence, and why some women choose to use the VR consistently, whereas others remove it temporarily or for longer periods, is important to determine the strategies required to improve adherence. In spite of the effectiveness of many PrEP products, their consistent use is behaviorally impacted, highlighting the importance of counselling messages. By understanding the reasons for non-adherence, counselling, education and messaging can be tailored and targeted to support correct and consistent VR use as it becomes available and accessible to the public. These messages and targeted support strategies could also inform the introduction of other multi-purpose prevention devices.

## Data Availability

Access to the data from this study may be requested through submission of a research concept to: cecilia.milford@gmail.com. The concept must include the research question, data requested, analytic methods, and steps taken to ensure ethical use of the data. Access will be granted if the concept is evaluated to have scientific merit and if sufficient data protections are in place.
